# Rectus Femoris Characteristics in Post Stroke Spasticity: Clinical Implications from Ultrasonographic Evaluation

**DOI:** 10.3390/toxins12080490

**Published:** 2020-07-31

**Authors:** Lucia Cosenza, Alessandro Picelli, Danila Azzolina, Marco Alessandro Minetto, Marco Invernizzi, Michele Bertoni, Andrea Santamato, Alessio Baricich

**Affiliations:** 1 Physical and Rehabilitation Medicine, Department of Scienze della Salute, University of Piemonte Orientale, 28100 Novara, Italy; marco.invernizzi@med.uniupo.it (M.I.); alessio.baricich@med.uniupo.it (A.B.); 2 Physical and Rehabilitation Medicine, “Maggiore della Carità” University Hospital, 28100 Novara, Italy; 3 Rehabilitation Unit, Department of Rehabilitation, “Santi Antonio e Biagio e Cesare Arrigo” National Hospital, 15121 Alessandria, Italy; 4 Neuromotor and Cognitive Rehabilitation Research Center, Department of Neurosciences, Biomedicine and Movement Sciences, University of Verona, 37134 Verona, Italy; alessandro.picelli@univr.it; 5 Department of Translational Medicine, University of Piemonte Orientale, 28100 Novara, Italy; danila.azzolina@uniupo.it; 6 Division of Physical Medicine and Rehabilitation, Department of Surgical Sciences, University of Turin, 10126 Turin, Italy; marco.minetto@unito.it; 7 Physical Medicine and Rehabilitation, ASST Sette Laghi, 21100 Varese, Italy; michele.bertoni@asst-settelaghi.it; 8 Physical Medicine and Rehabilitation Section, “OORR” Hospital, University of Foggia, 71122 Foggia, Italy; andrea.santamato@unifg.it

**Keywords:** muscle spasticity, botulinum toxin type A, ultrasonography, stroke, rehabilitation, stiff knee gait, rectus femoris

## Abstract

In stroke survivors, rectus femoris (RF) spasticity is often implicated in gait pattern alterations such as stiff knee gait (SKG). Botulinum toxin type A (BoNT-A) is considered the gold standard for focal spasticity treatment. However—even if the accuracy of injection is crucial for BoNT-A efficacy—instrumented guidance for BoNT-A injection is not routinely applied in clinical settings. In order to investigate the possible implications of an inadequate BoNT-A injection on patients’ clinical outcome, we evaluated the ultrasound-derived RF characteristics (muscle depth, muscle thickness, cross-sectional area and mean echo intensity) in 47 stroke survivors. In our sample, we observed wide variability of RF depth in both hemiparetic and unaffected side of included patients (0.44 and 3.54 cm and between 0.25 and 3.16 cm, respectively). Moreover, our analysis did not show significant differences between treated and non-treated RF in stroke survivors. These results suggest that considering the inter-individual variability in RF muscle depth and thickness, injection guidance should be considered for BoNT-A treatment in order to optimize the clinical outcome of treated patients. In particular, ultrasound guidance may help the clinicians in the long-term follow-up of muscle quality.

## 1. Introduction

Spasticity was first defined by Lance in 1980 as a motor disorder characterized by a speed-dependent increase in tonic stretch reflexes (muscle tone) with exaggerated tendon reflexes, secondary to hyperexcitability of the stretch reflex as a component of the upper motor neurons [1].

Hyperactivity of the rectus femoris (RF) due to spasticity can modify the gait pattern with a reduction of the peak knee flexion and consequent stiff knee gait (SKG) [2]. From a clinical point of view, SKG may result in altered foot clearance with an increased risk of falling [3]. Moreover, the compensation strategies to advance the limb in space, such as ipsilateral hip circumduction or contralateral vaulting, determine a less efficient gait pattern and increased energy expenditure [4].

Dynamic simulation studies showed that knee torque affects the swing phase more than hip torque, suggesting that inappropriate RF activity should reduce knee flexion [5].

The role of RF in SKG is also confirmed by the improvement in walking performance in terms of speed and maximum flexion of the knee in oscillation after motor branch block with anesthetic [6]. Botulinum toxin A (BoNT-A) represents the pharmacological treatment of choice for focal spasticity [7,8].

The action of BoNT-A consists of blocking neuromuscular transmission by inhibiting the release of acetylcholine at the neuromuscular junctions [9]. The significant benefits of treatment with BoNT-A are the reversibility of the effects and effectiveness in reducing hypertonic components with a low prevalence of complications [10]. U.S. Food and Drug Administration and the European regulatory agencies approved BoNT-A use for this indication [11].

BoNT-A injections have been proposed for the treatment in spastic RF, showing to improve the peak knee flexion in the swing phase of gait in stroke patients [12].

It should be pointed out that, in adults with focal spasticity, inaccurate muscle identification and poor injection technique are major causes for the loss of BoNT-A efficacy [10]. Based on these considerations, the accuracy of needle insertion is crucial in order to reach the BoNT-A treatment’s goal [13,14].

Nevertheless, to the best of our knowledge, instrument-guided injections of botulinum toxin are not routinely carried out [15] and anatomic landmark-guided needle placement is still the most commonly used BoNT-A injection technique for spasticity in adults [16]. It should be noted that no data are available concerning thickness of the anterior thigh subcutaneous tissue and RF characteristics in hemiparetic stroke survivors, and this critical issue can negatively affect a proper BoNT-A injection procedure in spastic RF.

Furthermore, the anatomic characteristics of the muscle can influence the treatment with BoNT-A and in the literature there are no data focusing on spastic RF features and quality.

Based on these considerations, the aim of this study is to evaluate the RF depth variability in stroke survivors affected by spasticity, based on ultrasonographic evaluations.

## 2. Results

Forty-seven patients were enrolled in the period between August 2018 and October 2019. Table 1 shows the demographic data referring to the sample under study. A total of 37 out of 47 subjects previously received BoNT-A injection in RF for SKG (non-naïve), whereas 10 did not (naïve).

Data were summarized according to groups as median and (25th–75th percentile), as shown in Table 2.

**Figure 1:**
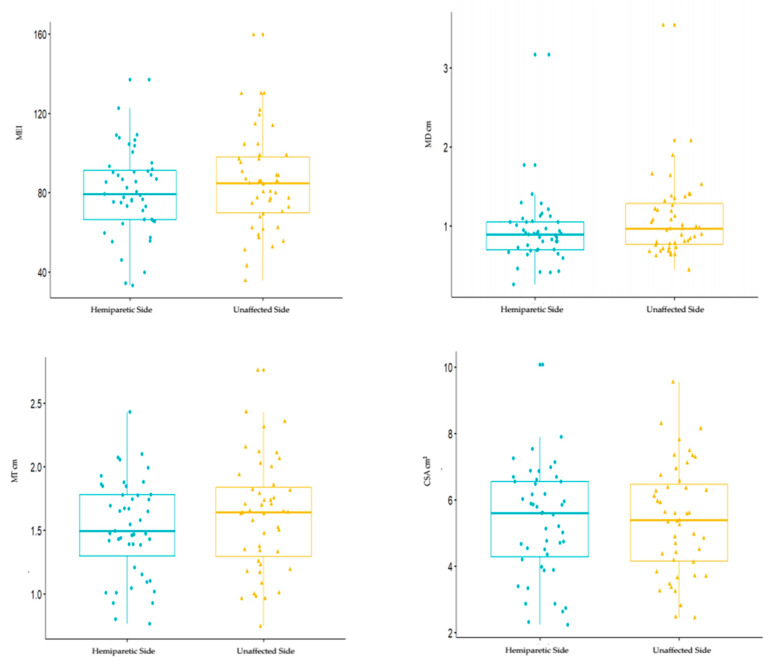
Boxplots of unaffected vs. hemiparetic sides MD, MT, CSA, MEI. Abbreviations: MD—muscle depth; MT—muscle thickness; CSA—cross-sectional area; MEI—mean echo intensity.

The comparison between unaffected and hemipareticsides did not show statistically significant differences, as shown in Table 2 and Figure 1.

In addition, comparison between treated and untreated hemiparetic sides did not show statistically significant differences, as shown in Table 3.

RF depth shows a statistically significant correlation with gender, as shown in Table 4: in males, the subcutaneous tissue is thinner and, consequently, the muscle is placed at a lower depth.

Both MT and CSA of RF show a statistically significant correlation with age and gender, both in the unaffected and hemiparetic side: in particular, MT and CSA decrease in older patients, assuming greater values in male subjects as depicted in Table 4.

## 3. Discussion

In our sample, we observed wide variability of RF depth in both hemiparetic and unaffected side of included patients (0.44 and 3.54 cm and between 0.25 and 3.16 cm, respectively), both in naïve and non-naïve patients.

In our sample of patients suffering from post-stroke spasticity, the physiological sexual differences in terms of reduced muscle mass and subcutaneous tissue depth in the female subjects are confirmed [17]

Additionally, in our sample age significantly affected muscle mass, as previously reported also in healthy subjects [18]

MD range observed in our patients, combined with inter individual physiological variability and possible muscle changes induced by spasticity, suggests that those aspects may severely affect the precision of BoNT-A injection, potentially reducing the clinical effect of BoNT-A treatment. Interestingly, Picelli et al. analyzed the accuracy of anatomic-landmark guided and electrical stimulation-guided injections in gastrocnemius muscle, measured using ultrasonography and found that the accuracy was significantly higher for the medial gastrocnemius than for the lateral, observing that the medial gastrocnemius was significantly thicker than lateral [19].

It should also be noted that current evidence suggests that proper needle placement is crucial in order to improve the clinical effect of BoNT-A, together with the reduction of side effects [20].

Picelli and colleagues observed an improvement in the clinical outcome after BoNT-A injections into the gastrocnemius of adults with spastic equinus with ultrasonography-guided injection technique, reporting that, one month after injection, both the modified Ashworth scale (MAS) and ankle passive range of motion improved better in the ultrasonography group than in the manual needle placement group [21].

Similar results were observed in the upper limb fingers and wrist flexor muscles, where clinical outcomes were significantly better in patients treated under ultrasound guidance [22,23] or electrical stimulation (ES) guidance than in patients injected using manual needle placement [23].

To the best of our knowledge, no published data are available to consider the impact of guided injection procedures for RF treatment with BoNT-A.

In a recent systematic review, the Authors strongly recommended an instrumented guidance of BoNT-A injection for the treatment of spasticity in adults and children (electrical stimulation- or ultrasound) and of focal dystonia such as spasmodic torticollis (electromyography) [24]. However, no specific recommendations can be made regarding the choice of instrumented guiding technique, except that ultrasound appears to be more effective than ES for spastic equinus in adults with stroke. Noteworthy, the Authors highlighted that studies are notably lacking for injection of the adductors, hamstrings and rectus femoris muscles.

In particular, the only available data to support the clinicians in identifying RF during BoNT-A injection were previously proposed for children with cerebral palsy; however, the Authors validated a protocol which requires a BoNT-A treatment under general anesthesia, and this procedure is not easily applicable in a clinical routine [25].

Considering the considerable MD and MT variability observed in this sample, our data support the current recommendations [24] for using guidance also for the injection in rectus femoris.

Moreover, from a routine clinical perspective, those aspects may also influence the injection procedure (e.g., needle size and length) in order to minimize the damage risk during the injection.

As previously stated, no data are available in order to clarify which guidance (e.g., ultrasound, electrical stimulation) should be preferable in clinical routine. It could be hypothesized that ultrasound guidance may help the clinicians in the long-term monitoring of spastic muscle quality. However, a recent survey reported that only 50% of clinicians are routinely supported by ultrasound during BoNT-A injection [26].

Additionally, another issue that should be considered is the potential muscle variation induced by spasticity (e.g., atrophy and/or fibrosis), that may influence the response to treatment with BoNT-A [27].

To investigate these aspects, we also analyzed the ultrasound muscle structure of RF, by comparing it with the same muscle on the untreated side in order to identify any changes in muscle quality.

As reported in the current literature, ultrasound is a valid and reliable tool for the evaluation of large muscles such as the quadriceps femoris in healthy young patients, but also in stroke outcomes [28]. Among the parameters that can be evaluated by ultrasound, MT, CSA and echogenicity of the muscle are easily available in real time and with low costs. Variations observed in these parameters, may reflect the presence of fatty infiltration and muscle fibrosis [29].

It should be noted that morphologic changes in muscle tissue in post-stroke spasticity can depend on several factors, such as disuse, denervation and atrophy [30].

Additionally, the role of BoNT-A treatment on muscle quality is still debated, and there are few and contradictory reports about this topic.

Some studies highlighted the negative effect of BoNT-A treatment, in particular on muscle size and morphology in patients affected by juvenile cerebral palsy (JCP) [31].

A peak of volume reduction was observed at 13 weeks from the injection and it remained at 25 weeks [32]. In another study, a reduction in gastrocnemius pennation angle after treatment with BoNT-A and an increase in fascicle length were observed in a sample of JCP patients; these data likely to be attributed to the reduction in spasticity induced by BoNT-A [33]. In both studies, the subjects enrolled were BoNT-A-naïve.

On the other hand, BoNT-A treatment was beneficial in reducing the stiffness of the calf muscle in JCP patients, with a proportional reduction in MAS [34].

Negative effects such as neurogenic atrophy of the medial gastrocnemius treated with BoNT-A have also been reported in healthy, adult volunteers [35].

Interestingly, no changes in muscle morphology were found following prolonged treatment with BoNT-A for blepharospasm [36]: these results may suggest that muscle quality is basically affected by spasticity, whereas an additional role of BoNT-A was not demonstrated in spastic muscles’ variations.

In our sample, the statistical analysis did not trace statistically significant differences between the two sides, and our data do not support the presence of muscle alterations induced by neither spasticity nor BoNT-A in spastic RF in stroke survivors.

To the best of our knowledge, this is the first study that includes data about muscle quality in spastic RF.

These results may have several explanations.

First, our sample included patients who were treated after a relatively short time after stroke, and this aspect may have reduced the risk of long-term changes in muscle architecture.

Moreover, other hypotheses may be based on the observations from BoNT-A use in esthetics, where BoNT-A use was speculated to reduce the risk of skin flap ischemia [37].

Thus, BoNT-A has also been tested in the muscle flaps and has proven effective in preventing muscle spasms without definitively denervating the flap. The transient effect of the toxin in the stabilization of muscle fibers reduces the risk of ischemia secondary to muscle spasm [38]. In in vitro studies, BoNT-A was shown to work by blocking norepinephrine and other vasoconstrictive substances in the arterial smooth muscle, thereby increasing the blood supply to the tissue in which it is injected [39]. This theory may help to explain the lack of muscle degeneration following repeated BoNT-A injection for spasticity treatment.

We are aware that this study has several limitations.

First, the sample size was small: this limitation may affect the generalizability of the observed features in the stroke survivor population.

Additionally, we analyzed a mixed population of naïve and non-naïve patients. Even if no significant differences were observed in muscle quality between these groups, this aspect may affect some characteristics of RF muscle.

## 4. Conclusions

Our findings show that considering the inter-individual variability in muscle depth and thickness of RF, injection guidance should be considered for BoNT-A treatment in order to optimize the clinical outcome of treated patients.

Additionally, available guidelines for muscle localization [40] and inoculation [14] may offer the chance to standardize injection procedures, in order to obtain homogeneous data on the clinical effect of BoNT-A treatment both in clinical and research settings.

Even if no data are available to clarify the optimal choice for RF injection, we may speculate that ultrasound could assist the clinicians in the long-term monitoring of muscle quality.

Further studies are needed in order to assess the impact of BoNT-A treatment on any changes in the rheological properties of muscle tissue in both naïve and non-naïve patients.

## 5. Materials and Methods

This was an observational study. Inclusion criteria were unilateral ischemic or hemorrhagic stroke (documented with clinical examination and neuroradiological findings), presence of spasticity at lower limb muscles equal to at least grade 1+ of the modified Ashworth scale and requiring medical treatment, age greater than 18 years. Exclusion criteria were inability to walk before the stroke, presence of cognitive impairment, presence of other musculoskeletal, neurological or cardiopulmonary impairment which can interfere with clinical findings and presence of skin lesions that can contraindicate BoNT-A treatment.

All participants were outpatients and gave their written informed consent for participation in the study, carried out according to the Declaration of Helsinki and validated by the local Ethics Committee (CE 158/20) and Competent Authority (Maggiore della Carità University Hospital, Novara, Italy Protocol13,989, validated on 15 June 2020).

**Figure 2:**
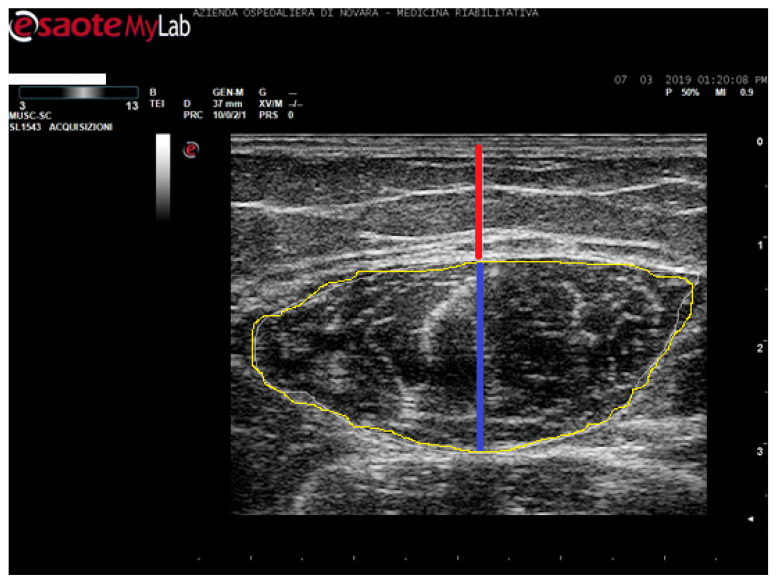
Real ultrasound images of RF. MD measurement is shown in red; MT measurement is shown in blue; CSA measurement is shown in yellow. MEI is calculated on the area circled in yellow using ImageJ.

Eligible patients for the study performed real-time B-mode ultrasonography using linear multifrequency transducer (scanning frequency 3–13 MHz, MyLab Six system, EsaoteSpA, Genoa, Italy). We placed the probe gently over the skin using water-soluble transmission gel in order to avoid compression or deformation of the muscle. The same physician took all measurements in order to avoid inter-individual variability. Images were acquired in transversal sections on both unaffected and hemiparetic sides, in the middle of the line that connects the anterior inferior iliac spine and the center of the patella where the cross-sectional area (CSA)of the RF is at maximum [41]. The collected images were stored and processed with ImageJ (National Institutes of Health, Bethesda, MD, USA) to measure the RF thickness as the distance between the superficial and the deep fascia at the widest distance; and the MD as the distance between the upper aponeurosis and the line separating the dermis from fat as shown in Figure 2 [42].

Moreover, CSA of the RF was analyzed and the software calculated mean echo intensity on the whole area (black = 0, white = 255) to track any changes in muscle tissue inoculated with BoNT-A.

Data were summarized to groups as median and [25th–75th percentile]. The differences between untreated and hemiparetic side were analyzed through a nonparametric Wilcoxon paired sample test. The difference between treated and untreated hemiparetic side were analyzed through a nonparametric Wilcoxon sample test.

The boxplots according to the untreated and hemiparetic side, are reported for MD (cm), MT (cm), CSA (cm^2^) and MEI.

An ordinary least square model was estimated adjusting muscle characteristics by age and gender. An interaction term of the side with the age and gender was also considered. A Huber-White [43] robust covariance estimation was considered for the analysis accounting for the within-side correlation.

A two-tailed test was considered for the hypothesis testing procedure and statistically significant values were considered to reach a *p*-value < 0.05. Statistical analyses were conducted using R 3.5.2 [44] with and regression modeling strategies (rms) [45] packages.

## Figures and Tables

**Table 1:** Demographic and clinical data of recruited patients (*n* = 47).

**Age (years) Mean (SD)** 25th–75th percentile	64 (10.87) 55/72
**Gender (male/female)**	28/19
**Time Stroke/1st Treatment (years)** 25th–75th percentile	2.7 0.22/2.26
**Time 1st Treatment/Acquisition(years)** 25th–75th percentile	3.4 0.22/6.31

Time elapsed between stroke and first treatment with botulinum toxin type A (BoNT-A) and time elapsed between first treatment with BoNT-A and ultrasound acquisitions.

**Table 2:** Descriptive statistics: unaffected vs. hemiparetic sides.

	Hemiparetic Side (*N* = 47)	Unaffected Side (*N* = 47)	Test
	25th–75th percentile Median	25th–75th percentile Median	Statistics
MD (cm)	0.76/1.28 1.08	0.69/1.05 0.92	*P* = 0.09
MT (cm)	1.29/1.84 1.62	1.30/1.78 1.52	*P* = 0.4
CSA (cm^2^)	4.15/6.47 5.41	4.28/6.55 5.33	*P* = 0.89
MEI	69.8/98.0 85.7	66.5/91.3 80.4	*P* = 0.46

Abbreviations: MD—muscle depth; MT—muscle thickness; CSA—cross-sectional area; MEI—mean echo intensity.

**Table 3:** Descriptive statistics: treated vs. untreated rectus femoris (RF).

	Hemiparetic Side Treated RF (*N* = 37)	Hemiparetic Side Untreated RF (*N* = 10)	Test
	25th–75th percentile Median	25th–75th percentile Median	Statistics
MD (cm)	0.79/1.35 1.11	0.69/1.17 0.97	*P* = 0.36
MT (cm)	1.34/1.82 1.63	1.18/2.00 1.56	*P* = 0.6
CSA (cm^2^)	4.18/6.37 5.45	3.70/6.46 5.26	*P* = 0.76
MEI	72.6/97.0 84.5	61.4/115. 4 89.8	*P* = 0.90

Abbreviations: MD—muscle depth; MT—muscle thickness; CSA—cross-sectional area; MEI—mean echo intensity.

**Table 4:** Age and gender ordinary least square (OLS) coefficients, with standard errors (SE) on the MD, MT and CSA outcomes. Interaction terms between age or gender with the side were also reported.

	Coefficient MD (cm)	S.E.	*p*-Value MD	Coefficient MT (cm)	S.E.	*p*-Value MT	Coefficient CSA (cm^2^)	S.E.	*p*-Value CSA
Age	0.0015	0.004	0.7281	-0.0139	0.003	<0.0001 *	−0.0493	0.013	0.0003 *
Gender (male)	−0.4313	0.132	0.0016 *	0.3109	0.087	0.0006 *	1.4918	0.386	0.0002 *
Age/side effect (unaffected)	−0.0035	0.003	0.1810	0.0105	0.056	0.0632	0.0184	0.88	0.3812
Gender/side effect (unaffected)	−0.0878	0.054	0.1071	0.0616	0.113	0.5865	−0.1085	−0.24	0.8083

Abbreviations: MD—muscle depth; MT—muscle thickness; CSA—cross-sectional area; S.E.—standard error. * Significant correlation (*p* < 0.05).

## Abbreviation

RF	rectus femoris
SKG	stiff knee gait
BoNT-A	botulinum toxin type A
MD	Muscle depth
MT	Muscle Thickness
CSA	Cross-sectional Area
MEI	Mean Echo Intensity
MAS	Modified Ashworth Scale
ES	electrical stimulation
JCP	juvenile cerebral palsy
